# Incessant ventricular tachycardia

**DOI:** 10.1007/s12471-016-0878-7

**Published:** 2016-08-25

**Authors:** R. Prisecaru, L. Riahi, Y. de Greef, D. Stockman, B. Schwagten

**Affiliations:** Cardiovascular Centrum Middelheim, Antwerpen, Belgium

## Answer

Among the side effects of adenosine, excitatory actions on ventricular automaticity enhanced by low concentrations of adenosine and the discharge of catecholamines are reported. The majority of adenosine-induced ventricular tachycardias (VTs) are rare, transient, not requiring further interventions and usually originate in the inferior left ventricular septum [1].

Since the coronary arteries showed no evidence of air embolism, thrombus or coronary atherosclerotic disease, adenosine-induced vasospasm is the most likely aetiology of the VT. The inferior ST elevation revealed in the two QRS complexes after the shock, together with the significant rise in troponin and the vasospasm depicted on angiography favour myocardial ischaemia and not a post cardioversion status [2]. The incessant character of the VT, the persistence of ST elevation and coronary vasospasm for more than just a few minutes after adenosine infusion and reversion following administration of nitrate may suggest a coronary hypersensitivity prone to develop spasm [3], such as Prinzmetal angina. Sympathetic nervous system activation is able to induce ventricular arrhythmia and coronary spasm by catecholamine release secondary to the pain triggered by the withdrawing of the transseptal sheath from the groin. This mechanism seems to be unlikely in the settings of general anaesthesia.

Although adenosine-induced incessant VT is a very rare arrhythmic complication during pulmonary vein isolation, its prompt recognition and treatment are of utmost importance. ECG monitoring is essential not only during adenosine infusion, but also during the recovery period in order to differentiate benign symptoms from serious complications such as VT and coronary spasm.
